# Attentional demand influences strategies for encoding into visual working memory

**DOI:** 10.2478/v10053-008-0007-2

**Published:** 2008-07-15

**Authors:** Jutta S. Mayer, Robert A. Bittner, David E. J. Linden, Danko Nikolić

**Affiliations:** 1 Laboratory for Neurophysiology and Neuroimaging, Department of Psychiatry, Johann Wolfgang Goethe-University, Frankfurt, Germany; 2 School of Psychology, University of Wales, Bangor, United Kingdom; 3 Frankfurt Institute for Advanced Studies, Johann Wolfgang Goethe-University, Frankfurt, Germany; 4 Max Planck Institute for Brain Research, Frankfurt, Germany

**Keywords:** attention, working memory, interference, encoding strategies

## Abstract

Visual selective attention and visual working memory (WM) share the same capacity-limited resources. We investigated whether and how participants can cope with a task in which these 2 mechanisms interfere. The task required participants to scan an array of 9 objects in order to select the target locations and to encode the items presented at these locations into WM (1 to 5 shapes). Determination of the target locations required either few attentional resources (“popout condition”) or an attention-demanding serial search (“non pop-out condition”). Participants were able to achieve high memory performance in all stimulation conditions but, in the non popout conditions, this came at the cost of additional processing time. Both empirical evidence and subjective reports suggest that participants invested the additional time in memorizing the locations of all target objects prior to the encoding of their shapes into WM. Thus, they seemed to be unable to interleave the steps of search with those of encoding. We propose that the memory for target locations substitutes for perceptual pop-out and thus may be the key component that allows for flexible coping with the common processing limitations of visual WM and attention. The findings have implications for understanding how we cope with real-life situations in which the demands on visual attention and WM occur simultaneously.

## Introduction

Visual selective attention and working memory (WM) are both concerned with the control of information, and both have limits with respect to how much information can be processed (Cavanagh & Alvarez, 2005; Duncan, Ward, & Shapiro, 1994; Luck & Vogel, 1997; Pashler, 1988; Phillips, 1974; Pylyshyn & Storm, 1988; Scholl, 2001). Moreover, studies suggest that visual attention and visual WM share to a high degree the same capacity-limited resources. To date, many studies have demonstrated interference between visual selective attention and visual WM (Awh & Jonides, 2001; Awh, Jonides, & Reuter-Lorenz, 1998; Barrouillet, Bernardin, Portrat, Vergauwe, & Camos, 2007; Jolicœur & Dell’Acqua, 1998, 1999; Oh & Kim, 2004; Smyth & Scholey, 1994; Woodman & Luck, 2004). For instance, a visual search task performed while spatial information was maintained in WM resulted in impaired search efficiency and impaired memory accuracy (Oh & Kim, 2004; Woodman & Luck, 2004). In addition, maintenance of information in spatial WM was incompatible with a secondary discrimination task when this task required shifts of spatial attention (Awh et al., 1998; Smyth & Scholey, 1994). Furthermore, visual selective attention was sensitive to interference from WM requirements in conditions of high memory load (de Fockert, Rees, Frith, & Lavie, 2001; Lavie, Hirst, de Fockert, & Viding, 2004). Finally, imaging studies have indicated that WM and attention tasks engage highly overlapping sets of brain regions (Corbetta, Kincade, & Shulman, 2002; LaBar, Gitelman, Parrish, & Mesulam, 1999; Pollmann & von Cramon, 2000) and that the activation patterns reflect competition for capacity-limited resources (Mayer et al., 2007).

If visual attention and visual WM share common resources and, thus, interfere when engaged simultaneously, the question is how these limitations can be overcome. An answer to this question should have relevance for many real-life situations. For example, while looking at a map and following the route between two locations, one might have to memorize the visual information needed to reach the destination, while at the same time using attention to search and navigate through the map. Thus, the aim of the present study was to investigate the strategies that allow participants to deal with such concurrent demands on visual selective attention and encoding into visual WM.

**Figure 1.**
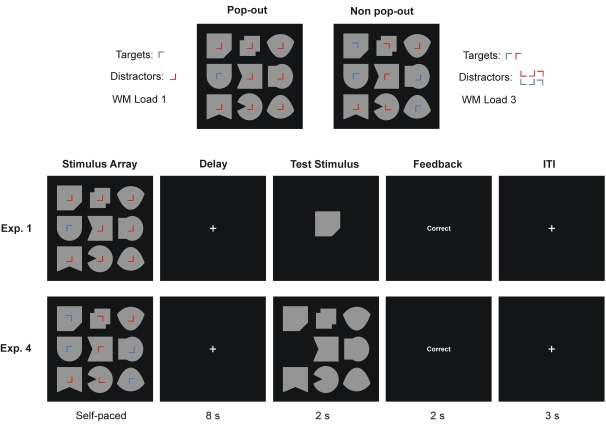
Examples of the stimuli and the procedures used in Experiments 1 and 4. Targets and distractors were distinguished by the items presented in the center of each object. Attentional demand for determination of the target locations was manipulated by the presence and absence of perceptual pop-out. In the pop-out condition blue target items were presented among red distractors. In the non pop-out condition colors were assigned randomly to the target items. Each stimulus array contained between one and five target items. In Experiment 1 participants determined the locations of the target items and memorized the shapes surrounding them whereas in Experiment 4 they memorized their locations only. The presentation time that was needed to achieve high WM performance was determined by the participants themselves. After an interval of 8 s, participants had to judge whether the test shape matched one of the target shapes (Exp. 1) or whether the location of the missing item in the test array matched one of the target locations (Exp. 4). ITI: Inter-trial interval.

Participants performed a task that combined the classical features of visual search experiments, which have been widely used in the study of selective attention (Treisman & Gelade, 1980; Wolfe 1998a), with those of visual WM studies (e.g., Oh & Kim, 2004; Olsson & Poom, 2005; Wheeler & Treisman, 2002). In each trial, participants were presented with an array of nine objects and had to memorize only some of them (targets), while the others could be ignored (distractors). Determination of the target locations was based on an L-shaped item located in the center of the object, but only the outer shape of the object and its orientation had to be remembered (see Figure 1). Thus, the present procedure allowed us to manipulate independently the demands on encoding into visual WM and the demands on attention for visual search of target locations. Attentional demand was manipulated by implementing two stimulation conditions in which the L-shaped items had either unique features (i.e., color) and were highly discriminable from the distractors (resulting in perceptual “pop-out” [PO]) or shared the features with the distractors and were thus difficult to discriminate (“non pop-out” [NPO]) (Duncan & Humphreys, 1989; Treisman & Gormican, 1988; Wolfe, 1998b). Only in the latter case did we expect that the determination of the target locations would require the attention-demanding serial search, which is commonly indicated by a linear increase in search times as a function of the number of distractor items in the array (Treisman & Gelade, 1980; Treisman & Sato, 1990). To manipulate the load of WM encoding, the number of target items was varied in each array, which ranged from one to five.

In the classical visual search paradigm, the display remains visible until the participant responds: Response accuracy is usually high. Therefore, response time (RT) is the most important measure in this paradigm as it indicates the amount of time required to determine the presence or absence of a target that is presented among distractors (Duncan & Humphreys, 1989; Treismann & Gelade, 1980; Treisman & Sato, 1990; Wolfe, 1998a, 1998b). This set-up was highly instrumental in the development of one of the most successful theories in psychology: the feature binding (feature integration) theory (Treisman, 1998; Treisman & Gelade, 1980; Treisman & Sato, 1990). In this paper the same concepts have been used to study the processes underlying the encoding of information into visual WM. Thus, the most important dependent variable was the presentation time of the stimulus array that participants needed to achieve good WM performance, and which they self-paced by a key press. We investigated how this time changed as a function of memory load and of attentional demand.

A similar dependent variable has been used in a recent study that investigated the role of visual WM for the formation of visual long-term memory (LTM, Nikolić & Singer, 2007). These authors first estimated the WM capacity for the locations of the target stimuli that either did or did not pop-out from the distractors, and then requested the participants to memorize accurately a number of target locations that grossly exceeded the capacity of WM. The participants self-paced the memorization process and the obtained encoding times were measured reliably (*r* > .90) and increased linearly as a function of target set size. Importantly, the changes in the slopes of these linear functions could be predicted accurately from the changes in the estimated WM capacities for the same stimuli. The authors concluded that the capacity of WM determined the speed with which visual LTM was created. This provided the missing evidence that visual WM played a pivotal role in the storage of information in visual LTM. Nikolić and Singer reported that the self-paced measure of the encoding times was reliable given that an immediate performance feedback was supplied at each trial, which, in turn, enabled the participants to learn quickly, on a trial-and-error basis, the minimum amount of effort (time) that was needed to achieve the required level of performance (95% correct in that study). In contrast, if such feedback was not provided, participants tended to shorten the encoding time and hence, trade the accuracy for speed. An important advantage of using the presentation time as a dependent variable in the present study was, similarly to the analyses conducted in the previous studies (Nikolić & Singer, 2007; Treisman & Gelade, 1980), that we could describe and analyze the data quantitatively by simple mathematical functions based on linear fits of differing intercepts and slopes.

Nikolić and Singer’s study (2007) investigated the WM capacity for the locations of the target stimuli only, thus without any additional contents presented on the display. In that study, WM could be loaded with very short stimulus presentations of about 1 s. In the present study we investigated the WM for relatively complex objects that were presented at the target locations. Thus, participants needed not only to select the target locations but also to extract and memorize the various shapes that were presented at these locations. This required a much longer presentation time than 1 s, as the information could not be loaded “directly” but successful encoding required the participants to engage into a more elaborated processing. The main goal of the present study was to investigate the nature of these processing steps, and to this end, two types of strategy were considered.

In a “search-and-encode strategy” participants encoded each shape as soon as they selected a relevant location, thus interleaving the search process with the WM encoding. In this case, presentation time should be simply divided between the two task components, and the presentation time that participants need in the non pop-out condition should be the sum of the presentation time in the pop-out condition and the time needed to select the relevant locations in the non pop-out condition. Thus, as empirical support for the search-and-encode strategy, we looked for evidence that the times for encoding and determination of target locations are additive.

The other considered strategy was postulated to involve two separate steps of encoding (“two-step encoding strategy”). In the first step participants selected and memorized only the locations of all target items and only then encoded the associated shapes at a later step. The additional process of memorizing the target locations requires additional processing time. For that case, a super-additive combination of the times for encoding and determination of target locations in the non pop-out condition was predicted. The time needed to memorize the locations was directly measured and whether this time corresponded to the additional time required to encode the target shapes in the non pop-out condition was investigated.

Importantly, the two-step encoding strategy but not the search-and-encode strategy implies interference between WM encoding and attention. A search-and-encode strategy should be possible if the two components need to be executed sequentially but do not interfere with each other, that is the search for a new target does not erase the contents stored previously in WM. As the existing evidence suggests that this is not the case (Awh et al., 1998; Barrouillet et al., 2007; Jolicœur & Dell’Acqua, 1998, 1999; Oh & Kim, 2004; Smyth & Scholey, 1994; Woodman & Luck, 2004), the two-step encoding strategy was considered as a possible tactic for overcoming this interference. Therefore, if empirical evidence favors one of the two strategies, the result also provides indirect information on whether, in this task, visual WM encoding and attention interfere.

### Synopsis of experiments

We conducted five experiments in which the study phase always consisted of identical stimuli, the tasks differing only in the instructions and in the test displays. Participants were debriefed at the end of each experiment and were asked about their subjective experience and strategies. In the main experiment (Experiment 1), participants encoded complex target shapes into WM, while determining their locations in a low or high attention-demanding visual search task (i.e., presence or lack of perceptual pop-out). WM performance was comparable across search conditions. Presentation time increased with increased WM load and, most importantly, with the lack of pop-out. Further experiments (Experiments 2 to 5) investigated the reason for the increase in the presentation time by contrasting the two, above described, strategies.

Experiment 2 and 3 tested the hypotheses of additivity versus super-additivity of the times needed to encode and determine the target locations. In Experiment 2, the time needed for simple visual search was measured. These times could not explain the increased presentation time produced by the lack of pop-out in Experiment 1. Therefore, Experiment 3 tested whether the slower processing in the non pop-out condition in Experiment 1 could be explained by repeated searches, owing to a putative lack of memory for visited target locations (Irwin, 1992; Peterson, Kramer, Wang, Irwin, & McCarley, 2001) and the need to search the entire array. The need to search repeatedly was reduced by informing the participants at each trial about the upcoming number of targets. The time saved by this manipulation again could not explain the costs on presentation time produced by the lack of pop-out in Experiment 1. Therefore, the results from Experiments 1 to 3 indicated consistently super-additivity of the times for encoding and determination of the target locations, favoring the two-step encoding strategy.

In the remaining two experiments (Experiments 4 and 5) the two-step strategy was tested further. The times were measured that participants needed to memorize the locations of the target items only and whether these times could explain quantitatively the difference between the pop-out and non pop-out conditions in Experiments 1 and 3 was investigated. Indeed, in Experiments 4 and 5, the times needed to memorize the target locations accounted well for the presentation time offsets between pop-out and non pop-out conditions in Experiments 1 and 3, respectively. These results again favored the two-step strategy.

## Experiment 1

Experiment 1 was used to investigate whether and how participants can encode complex objects into WM, while engaging selective attention for a visual search task. Participants memorized the shapes of only those objects whose center items matched the target items, and were instructed to ignore all the other objects. Determination of the target locations was easy in the pop-out condition and required attention demanding serial search in the non pop-out condition. WM for the shapes only was tested and there were no explicit requirements to use any particular strategy in this task. Thus, it was investigated whether participants could advance the WM performance in the non pop-out condition to the level of the performance in the pop-out condition, and if so, at what cost on presentation time.

### Method

#### Participants, apparatus, and stimuli

Thirty-six students and employees of the University of Frankfurt/M. (15 males, 21 females) volunteered for this study. The mean age of the participants was 26.1 years (range 19–33). In this and in all other experiments all participants reported normal or corrected-to-normal visual acuity, normal color vision, and no history of neurological or psychiatric illness.

The stimuli were presented through a PC on a 17-inch color monitor using ERTS (Experimental Run-Time System, Berisoft, Frankfurt, Germany). A chinrest was used to minimize head motion and to ensure that the observer’s eyes were positioned at a constant distance of 42 cm from the screen. Response keys were located on the computer keyboard. The experiments were performed in a dimmed room.

The display in the study phase consisted of nine different grey geometric shapes (each spanning approximately 1.1° × 1.1° of visual angle), arranged in a 3 × 3 matrix, and presented in the center of the screen and on a black background. The shapes were selected at random without replacement from a set of 12 shapes and each was oriented randomly in one of the four possible directions, so that in total it was necessary to discriminate between 48 different objects. In the center of each shape a small L-shaped item (0.3° × 0.3°) was placed. The Ls appeared in one of four different orientations (0, 90, 180, or 270°, clockwise) and were either blue or red in color (see Figure 1). Participants needed to memorize only the shapes associated with an L-oriented 90° (target items). The shapes associated with Ls of other orientations could be ignored (distractor items). The number of target items within each display varied randomly between one and five. In the pop-out condition target Ls always appeared in blue and distractors in red. Distractor Ls were always oriented at 270°. In the non pop-out condition each target and distractor was assigned randomly to either the color blue or red. In this condition, the distractor items could be any of the remaining three orientations (0, 180, and 270°). In the test phase participants were presented with a single shape in the center of the screen and without the center item. The luminance of the shapes, the blue, and the red center items was 12.3, 6.01, and 9.87 cd/m², respectively. The background luminance was 0.01 cd/m². During the delay period a white central fixation cross was presented on a blank screen (0.2° × 0.2°, 60.06 cd/m²).

#### Design and procedure

A 2 × 5 within-subjects factorial design was used, with two levels of attentional demand for determination of the target locations (pop-out and non pop-out) and five levels of WM load, determined by the number of targets (one to five targets). Each of the 10 experimental conditions was presented equally often (12 trials per condition). Pop-out (PO) and non pop-out (NPO) conditions were presented in separate blocks of 10 trials, with six blocks for each condition. This amounted to a total of 120 experimental trials per participant. The trials were fully randomized within blocks and pseudo-randomized across blocks and across participants. Before starting a new block, participants were always given instructions about the targets they needed to search for. At the beginning of the experiment participants performed two practice blocks of 10 trials, one for each of the two attentional conditions.

Each trial began with the presentation of the nine-item array, which remained visible until the participant pressed the response key. Participants had to determine the target locations and to memorize the shapes associated with the targets. The time they needed to achieve high memory performance, indicated by a key-press, was used as a dependent variable (presentation time). Participants were also instructed to place emphasis on accuracy over speed in order to ensure that response accuracy was high and comparable across different attentional-demand conditions. After the display disappeared participants fixated a cross during a delay period of 8 s, which was followed by the presentation of a single test shape. Participants were then required to indicate whether the test shape matched in form and orientation one of the target shapes presented previously by pressing the “Y” or “N” key for match and non-match, respectively. Half of the trials were matches. In 50% of the non-matches the probe stimuli differed with respect to the shape, and in the other 50% with respect to the orientation. The non-matches probe stimuli were selected from the set of all possible shapes that were not used as a target in a given trial. After each response feedback was given (“wrong”, “correct”, or “no response”), which was followed by an inter-trial interval of 3 s. Analyses of presentation time included only correct trials (see Figure 1 for an illustration of the sequence of events at each trial). The experimental procedure lasted approximately 60 min for each participant. After the experiment, participants were asked within a semi-structured interview freely to recall the strategies they used to accomplish the task. They were asked the following questions:

 1. What strategies did you use for searching the targets in the PO and NPO conditions? 

 2. What strategies did you use for encoding the objects in the PO and NPO conditions? 

 3. What strategies did you use for memorizing the objects in the PO and NPO conditions during the delay period? 

### Results and discussion

#### Accuracy at test

**Figure 2.**
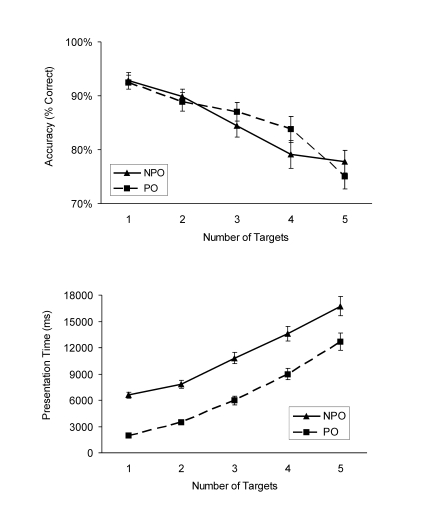
Results from Experiment 1. Mean response accuracy at test and mean presentation time as a function of number of targets and attentional demand (PO: pop-out, NPO: non pop-out). Vertical bars represent the standard error of the mean.

 Overall, response accuracy for the WM task was high (on average 85% correct) and decreased with the number of shapes that needed to be encoded – from 93% correct, with WM load 1 to 75% correct with WM load 5 in the pop-out condition, and from 93% correct with WM load 1 to 78% correct with WM load 5 in the non pop-out condition (see Figure 2, upper panel). These changes were significant, as tested by the main effect of number of targets in a 2 × 5 repeated measures ANOVA, *F*(4, 140) = 30.4, *p*< .001, η² = .47. Neither attentional demand nor the interaction between the two factors reached significance, *F*(1, 35) = 0.6, *p* = .46 and *F*(4, 140) = 1.8, *p* = .14, respectively. Given that response accuracy was high and comparable across the different levels of attentional demand, we concluded that the differences in the individually chosen presentation time indicated the differences in the processes required for successful WM encoding (see Presentation time section). According to Luck and Vogel (1997) , the load-dependent decrease in accuracy is likely to reflect the limited ability of maintaining information in visual WM rather than the limitations of the encoding process. Thus, this drop in performance should not have affected the processes of encoding information into WM, which was the main focus of our analyses. 

#### Presentation time

Participants were slower without than with perceptual pop-out and the presentation time increased with the number of targets that needed to be encoded (see Figure 2, lower panel). Repeated measures ANOVA, conducted with the same 2 ×5 design as for test performance, revealed significant main effects of attentional demand, *F*(1, 35) = 288.4, *p*< .001, η² = .892, and number of targets, *F*(4, 140) = 116.6, *p*< .001, η² = .769. The increase in presentation time as a function of number of targets could be explained very well by a linear approximation, and this was the case for both attentional-demand conditions (linear fits were *R*^2^ = .977 for pop-out and *R*^2^ = .983 for non pop-out). Quadratic models explained only 2.3% (pop-out) and 1.3% (non pop-out) of additional variance. Therefore, the subsequent analyses of these data were made on the basis of linear approximation. On average, participants needed 2706 ms for encoding into WM each additional target shape in the presence and 2606 ms in the absence of perceptual pop-out. The relatively slow rates of these linear functions indicated that the process of encoding complex shapes into WM was difficult and already capacity-demanding in the pop-out condition.

Importantly, the interaction between attentional demand and the number of targets was not significant, *F*(4, 140) = 1.2, *p* = .32, indicating that the slopes relating the average presentation time to the number of targets were practically identical in the two attentional-demand conditions. The offset between the two slopes, that is the difference between non pop-out and pop-out conditions, ranged between 4008 and 4853 ms with an average of 4490 ms (see Table 1). Thus, the manipulation of attentional demand added considerable processing time but this time was constant across the number of targets. This result indicates that the manipulation of attentional mechanisms produced an effect on presentation time that was independent of the effect produced by the manipulation of WM load. Therefore, the results from Experiment 1 suggest that participants can achieve high memory performance despite the lack of pop-out but that this comes at the price of longer presentation time.

**Table 1.** Offsets (i.e., differences) in presentation time (Experiments 1, 3, 4, and 5) and counting time (Experiment 2) between non pop-out and pop-out conditions across WM (working memory) loads 1 to 5.

WM load	Experiment 1	Experiment 2	Experiment 3	Experiment 4	Experiment 5
1	4644	2897	2145	3848	1721
2	4339	2955	2565	4024	2279
3	4853	2980	3554	4085	3062
4	4611	2900	3937	3800	3293
5	4008	2852	3554	3993	3563
Mean	4490	2917	3151	3950	2784

#### Reported encoding strategies

The majority of participants (32 of 36) reported that in the non pop-out condition they needed to use a two-step encoding strategy: In the first step they selected and memorized the locations of all the target items, encoding the associated shapes only in the second step. Three participants reported using a search-and-encode strategy in the non pop-out condition, encoding each target shape immediately after selecting a target item and making only one sweep through the array. One participant did not report any specific strategy.

We found no significant differences in response accuracy and presentations times between participants subscribing to different encoding strategies, *F*(1, 33) = 0.3, *p* = .88 for presentation time, *F*(1, 33) = 0.06, *p* = .82 for accuracy. However, due to vastly unequal numbers of participants in the two groups (32 vs. 3 participants) this result should be taken with caution.

## Experiment 2

In this experiment we investigated whether the offset in presentation time between the two attentional-demand conditions observed in Experiment 1 could be explained by visual search for target locations. To estimate the time to select target locations in this task, we presented the same stimuli as in Experiment 1 but asked participants to count only the number of target items in the array. This task required engagement of attention for determination of the targets, but not the processing of the background shapes, nor did it pose any demands on WM for shapes. Participants were again instructed to place the emphasis on accuracy over speed in order to ensure that the criteria for determination of the target locations were similar to those in Experiment 1. If the offset in the presentation time between pop-out and non pop-out conditions in Experiment 1 was due to the attention-demanding visual search, a similar offset should be found between pop-out and non pop-out conditions in the counting times.

### Method

#### Participants, apparatus, stimuli, procedure, and design

Fourteen students and employees of the University of Frankfurt/M. (6 males, 8 females) participated in this study. Their mean age was 26.7 years (range 19–44). Five of the participants had also taken part in Experiment 1.

Participants were required to count the target items in the same stimulus array as used in Experiment 1. After completing the count, participants indicated the search time by pressing the “return” key on the computer keyboard. After this key-press a question mark appeared in the center of the screen that prompted the participants to enter the number of the counted targets. Participants were instructed to place the emphasis on accuracy over speed during the counting process and were informed that the time needed to enter the counted number of targets was irrelevant. After each response, the question mark disappeared and feedback (“wrong”, “correct”, or “no response”) was provided and followed by an inter-trial interval of 3 s. Only correct trials were included in the analyses of counting times. The experimental proce

dure lasted approximately 30 min for each participant.

We used a 2 × 5 within-subjects factorial design with two levels of attentional demand for determination of the targets (pop-out and non pop-out) and five different counts (one to five targets).

### Results and discussion

#### Accuracy at test

**Figure 3.**
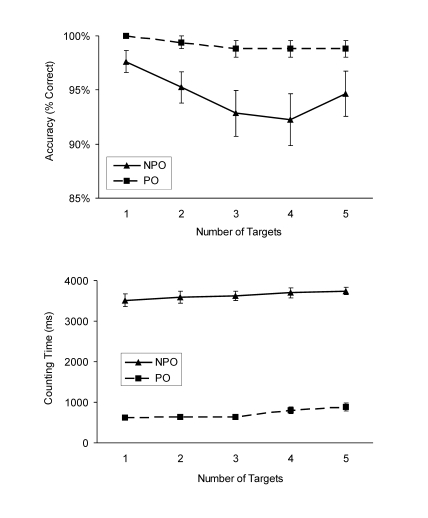
Results from Experiment 2. Mean response accuracy at test and mean counting time as a function of number of targets and attentional demand (PO: pop-out, NPO: non pop-out). Vertical bars represent the standard error of the mean.

Overall, response accuracy was high (on average 97% correct). A repeated measures ANOVA revealed only a significant main effect of attentional demand, *F*(1, 13) = 32.4, *p*< .001, η² = .71, and neither the number of targets nor the interaction between the two factors reached significance, *F*(4, 52) = 1.6, *p* = .21, and *F*(4, 52) = 0.7, *p* = .54, respectively. Participants counted target items less accurately in the non pop-out (on average 94.4% correct) than in the pop-out conditions (on average 98.9% correct) (see Figure 3, upper panel). In the non pop-out condition the errors were more often underestimates (about 86%) than in the pop-out condition (about 66%), indicating that the increase in the similarity between targets and distractors increased the probability that a target item would be missed.

The accuracy in the non pop-out conditions of the present task was higher than in a control version of the same task in which participants were instructed to place the emphasis on speed over accuracy (90.6% correct vs. 94.4% correct, *t*(22) = 2.16, *p*< .05; other results are not shown for the control experiment). Therefore, the results from the present task, in which accuracy was emphasized, indicate that participants followed this instruction. Thus, any increase in counting times in the non pop-out compared with the pop-out condition should be attributed to slower perceptual processing and should not be influenced by changes in speed–accuracy tradeoff across different perceptual conditions.

#### Counting time

Participants were slower in the non pop-out compared with the pop-out condition, and counting times increased linearly with the number of targets (linear fits were *R*^2^ = .865 for pop-out and *R*^2^ = .991 for non pop-out; see Figure 3, lower panel). Participants needed on average 72 ms for counting each additional target item in the presence and 57 ms in the absence of perceptual pop-out. A repeated measures ANOVA revealed significant main effects of attentional demand, *F*(1, 13) = 1292, *p*< .001, η² = .99, and number of targets, *F*(4, 52) = 8.4, *p*< .001, η² = .39, but the interaction between attentional demand and number of targets was not significant, *F*(4, 52) = 0.4, *p* = .81. Accordingly, the offset produced by the non pop-out condition compared with the pop-out condition was almost constant across the number of targets and was on average 2917 ms, range 2852–2980 ms (see Table 1).

The similarity of the two slopes relating the counting time to the number of targets indicates that these slopes mostly reflect the time needed to perform counting operations, such as the verbal act of increasing the counter by one upon the selection of the target, and thus, that these operations are not directly related to visual search. Visual search processes should be reflected solely in the described offset in the counting times because participants always needed to search the entire arrays, regardless of the number of targets. In order to estimate the rate of this search, it was necessary to take into account the constant processing time that was not related to the sequential component of the search process (i.e., the intercept). Although this time could not be directly measured from the present data, it was assumed that this time largely corresponded to the counting times in the non pop-out condition. Thus, the search rate in the non pop-out condition was estimated simply by taking the mean offset of counting times between the two attentional-demand conditions and dividing this number by the number of elements in the array (nine). This resulted in a time period of 324 ms to scan each of the nine locations. Although this time period is higher than the search rates reported in standard inefficient visual search tasks (Duncan & Humphreys, 1989; Treisman & Gormican, 1988; Wolfe, 1998b), it is consistent with reports that search time increases with the complexity of the items (Alvarez & Cavanagh, 2004). The slower search speed in our task than in standard visual search tasks cannot be simply explained by the need to select and count multiple targets because such tasks do not produce similar increases in response times (Horowitz & Wolfe, 2001). It can also be excluded that the prolonged search time was a result of the instruction to emphasize accuracy because, in one control experiment (not reported here), we instructed 10 participants to count the target items as quickly as possible and obtained only slightly faster search times (280 ms to scan each of the nine locations). Another reason why visual search was so slow in the present experiment might be that attention tends to be locked onto perceptual objects. When attention is voluntarily placed upon one feature of an object it automatically spreads to other features of the same object (Duncan, 1984; Scholl, 2001; Vecera & Farah, 1994). Thus, when attention was placed on the features defining the targets in the present task, the attentional spotlight may have tended to spread over the other features of the objects, making it more difficult to scan multiple items simultaneously and/or judge whether this item was a target.

The important finding for the present study is that the offsets in the counting time between pop-out and non pop-out conditions (on average 2.9 s) were smaller in the present experiment than the offsets in the presentation time in Experiment 1 (on average 4.5 s, see Table 1). These differences were statistically significant, *F*(1, 48) = 13.4, *p*< .01, η² = .22. We also tested whether this comparison might have been confounded by a perceptual learning effect that could have occurred in the 5 participants who also took part in Experiment 1. A comparison between the 9 new and 5 old participants revealed no significant effect of the factor task exposure (new vs. old participants), and neither were the interactions of this factor with the factors attentional demand or WM load significant (repeated measures ANOVA, all F-values < 0.6, all p-values >.57). Therefore, it was concluded that serial search accounted for only about two thirds of the processing costs that arose due to the lack of pop-out in Experiment 1. These findings suggest a super-additive increase in the times for encoding and determination of target locations in the non pop-out condition, which is consistent with the idea of interference between attention and visual WM encoding. However, it is first investigated in Experiment 3 whether the remaining one third (or about 1.6 s) of the offset between pop-out and non pop-out conditions could be explained by repeated serial searches.

#### Reported search strategies

In the non pop-out condition all participants reported scanning the array serially, mostly from the upper left corner towards the lower right, and making one single sweep through the array. In the pop-out condition participants reported detecting the target items at a glance.

## Experiment 3

The aim of this experiment was to assess whether repeated searches could explain the difference between the presentation time of the non pop-out and pop-out conditions of Experiment 1. Several studies have demonstrated that the temporary storage of previously searched target locations decays over time (Irwin, 1992; Phillips, 1974) and that participants sometimes need to repeat the search at target locations that they have already visited previously (Peterson et al., 2001). Repeated searches might have occurred in the non pop-out condition of Experiment 1 because (a) multiple targets were presented, (b) participants had to perform a difficult additional task of encoding information into WM, and (c) participants always needed to scan the entire array, even when there was only one target. This was because they did not know how many targets would be presented at a given trial.

To assess the degree to which the lack of knowledge about the number of targets contributed to the time offsets between pop-out and non pop-out conditions and therefore, to assess the extent of possible repeated searches, participants in Experiment 3 were informed about the upcoming number of targets prior to each experimental trial. This manipulation was expected to reduce the presentation time in the non pop-out conditions, especially with a small number of targets (one or two targets). The main question then was whether this reduction would explain all of the differences between the search time, as determined from Experiment 2, and the presentation time in the non pop-out condition of Experiment 1. In this case it could be concluded that repeated searches explained the non-pop-out offset in Experiment 1. This finding would support a search-and-encode strategy. Conversely, if an offset between pop-out and non pop-out conditions remained even in Experiment 3, where the number of targets was known beforehand, this would suggest that a particular cognitive process supporting WM encoding placed a particular demand on presentation time. We would suggest that it is the process of memorizing all target locations.

A second question addressed by Experiment 3 concerned the role of verbal coding. The phonological store is highly efficient for serial recall, thus participants tend to recode visually presented items into a verbal code (Baddeley, 2000). Indeed, in Experiment 1, the majority of participants reported creating their own verbal labels for the complex shapes. As the aim of the present study was to investigate visual attention and WM, it was necessary to assess the role of verbal encoding during the encoding of the shapes into WM. To this end, an articulatory suppression task was implemented that is known to reduce, albeit not completely eliminate, subvocal rehearsal and the phonological encoding of visually presented material (e.g., Baddeley, 2000, 2003; Besner, Davies, & Daniels, 1981; Murray, 1968). If presentation time and accuracy did not substantially differ between Experiment 1 without articulatory suppression and Experiment 3 with articulatory suppression, it could be concluded that the encoding and storage of complex shapes depends to a high degree on visual processing of information.

### Method

#### Participants, apparatus, stimuli, procedure, and design

Sixteen students and employees of the University of Frankfurt/M. (7 males, 9 females) participated in this experiment. The mean age of the participants was 24.6 years (range 18–44). Six participants also took part in Experiment 2, only one of them took part in Experiment 1.

The stimuli, procedure, and design were the same as in Experiment 1, apart from the following two differences. First, at the beginning of each trial a digit was presented at the center of the screen, for 2 s. This digit indicated the number of target items that would be presented in the upcoming stimulus array. Second, the articulatory suppression task required participants to repeat aloud a syllable la throughout the duration of the trial.

### Results and discussion

#### Accuracy at test

**Figure 4.**
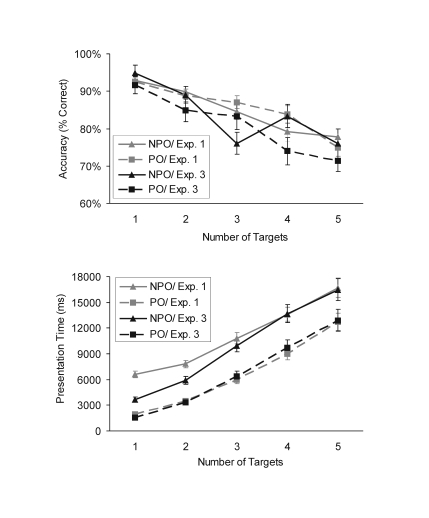
Results from Experiment 3 compared with the results from Experiment 1. Mean response accuracy at test and mean presentation time as a function of number of targets and attentional demand (PO: pop-out, NPO: non pop-out). Vertical bars represent the standard error of the mean.

A repeated measures ANOVA revealed a significant main effect of number of targets, *F*(4, 60) = 13.4, *p*< .001, η² = .47, but no effect of attentional demand, *F*(1, 15) = 2.8, *p* = .12. The interaction between the two factors also reached significance, *F*(4, 60) = 3.3, p < .05, η² = .18, but the averages did not show any consistent relationships between the variables (see Figure 4, upper panel) and explained only 18.1% of the variance in the dependent factor. Therefore, this interaction was not used for further interpretation of the results.

These results are highly consistent with those observed in Experiment 1, showing that response accuracy decreases with the number of targets to be remembered but does not depend on the attentional demand condition. Also, participants were about as equally accurate as they were in Experiment 1 (on average 82% correct, range 71–95%, in Experiment 3; on average 85% correct, range 75–93%, in Experiment 1) and there were no significant differences between these two experiments, *F*(1, 50) = 1.5, *p* = .14, for pop-out, *F*(1, 50) = 0.7, *p* = .46, for non pop-out. These results indicated that articulatory suppression did not affect participants’ ability to memorize the shapes. This finding suggests that in the present task it was not necessary to recode the visual information into a verbal form in order to achieve good memory performance. This conclusion was also supported by the presentation time data (see next section).

#### Presentation time

Similarly to Experiment 1, participants were slower in the non pop-out than in the pop-out condition, *F*(1, 15) = 127.9, *p*< .001, η² = .89. Presentation time also increased linearly with the number of targets that needed to be encoded into WM in both the pop-out and the non pop-out conditions (linear fits were *R*^2^ = .989 for pop-out and *R*^2^ = .992 for non pop-out), and these increases were significant, *F*(4, 60) = 70.4, *p*< .001, η² = .82 (see Figure 4, lower panel). The slope relating the average presentation time to the number of targets was steeper for non pop-out (3338 ms) than for pop-out (2918 ms), leading to a significant interaction between attentional demand and number of targets, *F*(4, 60) = 4.8, *p*< .01, η² = .24. In the pop-out condition, these slopes were not significantly different from Experiment 1, *t*(50) = 0.53, *p* = .60, but in the non pop-out condition the average difference of 732 ms approached statistical significance, *t*(50) = 1.67, *p* = .10. The offset in the presentation time between pop-out and non pop-out conditions increased from 2145 ms, for one target to 3554 ms, for five targets (see Table 1). Thus, as predicted, the presentation time was reduced in the non pop-out conditions with smaller numbers of targets as compared with the presentation time in Experiment 1 (in particular with one and two targets, see Figure 4) . With the memory loads four and five presentation time was indistinguishable across the two experiments, *t*(50) = 0.13, *p* = .90, and this was the case for each number of targets in the pop-out condition, *F*(1, 50) = 0.04, *p* = .85[Fn FN1].^1^

**Figure 5a and 5b.**
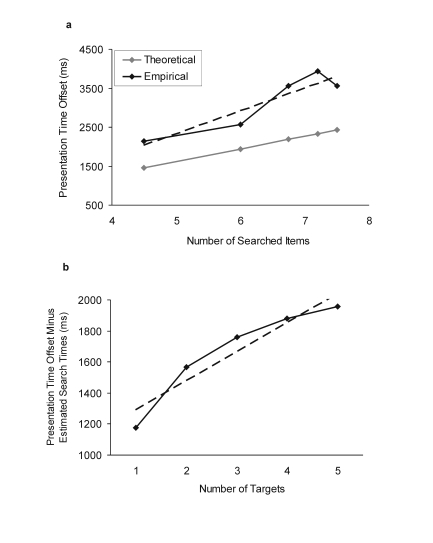
Figure 5a. Empirically obtained offset in the presentation time produced by the lack of pop-out in Experiment 3 and theoretically predicted offset assuming a single-sweep search. X-axis presents the average numbers of items that needed to be searched if one to five targets are presented in the array. Dashed line illustrates linear fit (parameters reported in the main text). Figure 5b. The difference between the two offsets in Figure 5a, expressed as a function of number of target items. Dashed line illustrates linear fit (parameters reported in the main text).

We next investigated whether the presentation time in the non pop-out condition equaled the sum of the encoding time in the pop-out condition plus the time needed to select the target location(s) by a single-sweep search. If this was the case for any of the five memory loads, evidence would be provided that, for that load condition, participants first searched and then immediately encoded the information into WM. To conduct this analysis, first the expected number of array items was estimated that needed to be searched for the presence of a target at each WM load (k) which, if the targets are positioned randomly, is given by the following equation (*A* and *N* represent the array size and the number of targets, respectively): *k* = *A* – *A* / (*N* + 1). For *N* = [1, 2, 3, 4, 5] in an array of *A* = 9, the expected numbers of items searched were 4.5, 6, 6.75, 7.2, and 7.5. These values were then multiplied by the expected search time per single item, which according to the results from Experiment 2, was 324 ms. The resulting theoretical values are plotted in Figure 5a together with the offset in the presentation time between non pop-out and pop-out conditions obtained empirically. It can be seen that the theoretical and empirical values do not match. The empirical offset in the presentation time was, already with WM load 1 (i.e., 4.5 items searched), considerably larger than that predicted by a single-sweep search. This difference increased further with the higher WM loads as the slope with which the empirical values increased was much steeper than expected by simple search for target items (585 vs. 324 ms, 81% higher slope, linear fit *R*^2^ = .86). The difference between the two, expressed as a function of the number of target items, accumulated to over 1.8 s with WM load 5 (see Figure 5b) whereas the large positive intercept of the resulting function (slope 188 ms, intercept 1104 ms, linear fit *R*^2^ = .903) indicated that with the lack of pop-out participants needed a constant time of 1104 ms irrespective of the number of targets. These results suggest that simple serial search does not account for the slowdown in the presentation time caused by the lack of pop-out even when the participants know the number of targets presented in the array. This result holds for all five memory load conditions.

Taken together, the results of Experiments 1 to 3 indicate an excess in the costs on presentation time produced by the lack of perceptual pop-out, and this cost cannot be explained fully by simple visual search or by repeated searches for targets. Thus, the presentation time does not simply represent a sum of the two task components and therefore is not consistent with a search-and-encode strategy that interleaves the search process with the WM encoding. Instead, the results revealed a super-additive increase of the times for encoding and determination of target locations, indicating that participants used another, time-consuming strategy. One possibility, as suggested by the finding that WM and attention interfere (Awh et al., 1998; Barrouillet et al., 2007; Jolicœur & Dell’Acqua, 1998, 1999; Oh & Kim, 2004; Smyth & Scholey, 1994; Woodman & Luck, 2004), as well as by the subjective reports of our participants, is that they invested the additional time in the process of memorizing all target locations prior to encoding their shapes. This two-step strategy was investigated more directly in Experiments 4 and 5.

#### Reported encoding strategies

All 16 participants reported using the same two-step strategy as described by the majority of participants in Experiment 1.

## Experiment 4

In Experiments 4 and 5 we explicitly tested the strategy that was reported by the participants during the debriefing procedure. The majority of participants reported that, in the non pop-out condition of Experiments 1 and 3, they memorized first the locations of all the targets and only then did they encode the shapes of the associated objects. To search for experimental evidence supporting this claim we presented participants with the same stimuli as in Experiment 1 but asked them to memorize the locations of the target items only. If participants used the reported strategy, the time they needed to search and memorize the target locations (e.g., the offsets in the presentation time between non pop-out and pop-out conditions) should correspond to the presentation time offsets between non pop-out and pop-out conditions in Experiment 1.

### Method

#### Participants, apparatus, stimuli, procedure, and design

Sixteen students and employees of the University of Frankfurt/M. (8 males, 8 females) participated in this experiment. The mean age was 27.1 years (range 19−39). Eight participants took part in Experiment 1 and 2 of them also took part in Experiment 2.

The stimuli, procedure, and design were the same as those in Experiment 1, apart from the following two differences. Participants were instructed to determine and memorize the locations of the target items only and thus to ignore the shapes of the associated objects. In order to probe WM for target locations, the original stimulus array was presented at the test phase without the center items and with one of the shapes missing. Participants needed to indicate whether the location of the missing shape matched one of the target locations.

After each response feedback was given (see Figure 1).

### Results and discussion

#### Accuracy at test

**Figure 6:**
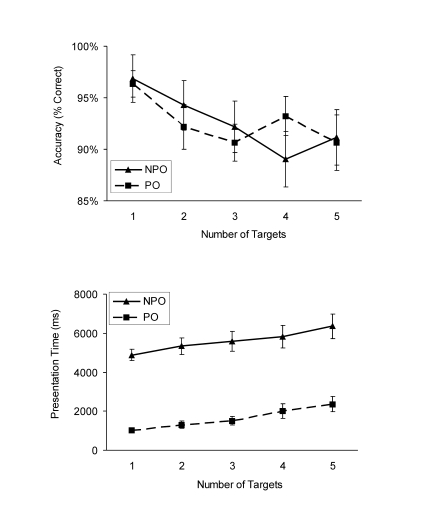
Results from Experiment 4. Mean response accuracy at test and mean presentation time as a function of number of targets and attentional demand (PO: pop-out, NPO: non pop-out). Vertical bars represent the standard error of the mean.

Overall, response accuracy was again high (on average 93% correct). A repeated measures ANOVA revealed only a significant main effect of number of targets, *F*(4, 60) = 5.8, *p*< .01, η² = .27. Neither attentional demand nor the interaction between the two factors was significant, *F*(1, 15) = 0.01, *p* = .96, and *F*(4, 60) = 0.7, *p* = .57, respectively. Thus, similar to Experiment 1, response accuracy decreased with the number of targets whose locations needed to be encoded and, again, did not differ between pop-out and non pop-out conditions (see Figure 6, upper panel). Participants responded more accurately in Experiment 4 than in Experiment 1, *F*(1, 50) = 9.8, *p*< .01, η² = .16 for pop-out and *F*(1, 50) = 10.6, *p*< .01, η² = .18 for non pop-out (on average 93% correct, range 89–97% in Experiment 4; on average 85% correct, range 75–93 % correct in Experiment 1), indicating that their memory for locations was better than their memory for shapes. The eight participants who took part in Experiment 1 were no more accurate than 8 new participants. Instead, it was the new participants who tended to be more accurate (95% vs. 90% correct); however, the difference did not reach the level of significance, *F*(1, 14) = 4.2, *p* = .06, η² = .23. Also, task exposure did not interact with attentional demand or WM load (repeated measures ANOVA; all *F*-values < 2.1, all *p*-values > .12). Therefore, this experiment did not produce any evidence that improvement due to perceptual learning had taken place among participants who took part in multiple experiments of the study.

#### Presentation time

Similarly to Experiment 1, participants were slower without than with perceptual pop-out, *F*(1, 15) = 193.9, *p*< .001, η² = .93. Presentation time increased linearly with the number of targets that needed to be encoded into WM, in both, the pop-out and non pop-out conditions (linear fits were *R*^2^ = .976 for pop-out and *R*^2^ = .978 for non pop-out); these changes were significant, *F*(4, 60) = 11.2, *p*< .001, η² = .43 (see Figure 6, lower panel). The interaction between attentional demand and number of targets was not significant, *F*(4, 60) = 0.5, *p* = .71, again indicating almost identical slopes relating the average presentation time to the number of targets across the two levels of attentional demand.

The slopes were much shallower in the present experiment than in Experiment 1. On average, participants needed 342 ms to encode each additional location of a target item in the absence and 336 ms in the presence of perceptual pop-out (compared with 2606 and 2706 ms for encoding shapes in Experiment 1). Thus, locations were encoded much faster than shapes. A repeated measures ANOVA with the factors attentional demand, WM load, and task exposure (new vs. old participants) revealed no significant effect either for the factor task exposure or for its interaction with the other two factors (all F-values < 1.1, all p-values > .31). Thus, again no evidence was found that improvement due to perceptual learning had taken place among the eight participants who also took part in Experiment 1.

Similarly to Experiment 1, the offsets between pop-out and non pop-out conditions were practically constant across different WM loads. Although the offsets were smaller in magnitude compared with those in Experiment 1 (*M* = 3950 ms, range 3800–4085 ms in Experiment 4; compared to *M* = 4490 ms, range 4008–4853 ms in Experiment 1) these differences were not significant, *F*(1, 50) = 1.5, *p* = .23 (see Table 1). Thus, the results indicate additivity between the presentation time in the pop-out condition of Experiment 1 and the time offset between pop-out and non pop-out conditions in Experiment 4. In other words, when the time needed to encode the shapes is taken into account, the lack of pop-out caused similar effects on presentation time in Experiments 4 and 1. Therefore, the time needed to memorize the locations seems to be a reasonable explanation of the time offset between pop-out and non pop-out conditions in Experiment 1.

#### Reported encoding strategies

The majority of participants (15 of 16) reported integrating the target locations into one or two perceptual representations that could be described either as a spatial template, a shape composed of the individual locations, or as a chunk. One participant reported encoding discrete locations, one after another, without a particular perceptual organization.

## Experiment 5

When informed about the upcoming number of targets in Experiment 3, participants also reported using a two-step strategy. Apparently, they memorized the locations of all targets first and only then encoded the shapes into WM. These reports, together with the results of Experiment 4, suggest that if participants are informed about the number of target locations, the times needed to memorize those locations might explain the peculiar offsets in the presentation time between pop-out and non pop-out conditions found in Experiment 3. Therefore, in Experiment 5, participants were informed prior to each trial about the number of target items in the upcoming stimulus array, as in Experiment 3, and asked to remember the locations of the targets only, as in Experiment 4. The analysis was similar to that used in Experiment 3.

### Method

#### Participants, apparatus, stimuli, procedure, and design

Ten students and employees of the University of Frankfurt M. (4 males, 6 females) participated. The mean age was 25.2 years (range 20−33). None of the partici-pants took part in any of the previous experiments.

The stimuli, procedure, and design were the same as in Experiment 4, apart from the following two differences. First, the procedure from Experiment 3 was used to inform participants about the number of upcoming targets at the beginning of each trial. Second, the articulatory suppression task was implemented.

### Results and discussion

#### Accuracy at test

As in the previous experiments, response accuracy was high at both levels of attentional demand (on average 94 % correct), decreased as a function of WM load, *F*(4, 36) = 3.3, *p*< .05, η² = .27, but did not depend on the attentional-demand condition, *F*(1, 9) = 0.4, *p* = .58. As in Experiment 3, the interaction between the two factors was also significant, *F*(4, 36) = 3.3, *p*< .05, η² = .27 (graph not shown). Response accuracy in this experiment did not differ from that obtained in Experiment 4, *F*(1, 24) = 0.8, *p* = .39, for pop-out; *F*(1, 24) = 0.5, *p* = .83, for non pop-out (on average, 94% correct, range 84–99% correct, in Experiment 5; on average, 93% correct, range 89–97% correct, in Experiment 4). As in Experiment 3, the finding that articulatory suppression did not impair participants’ ability to memorize the locations indicates that the memory of locations was based, to a high degree, on visual processing. This conclusion was further supported by the lack of significant differences between the presentation time obtained in Experiments 5 and 4 (see Presentation time section).

#### Presentation time

**Figure 7a and 7b.**
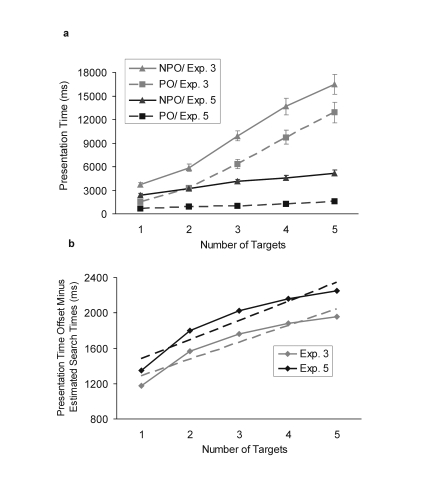
Results from Experiment 5 compared with the results from Experiment 3. Figure 7a. Mean presentation time as a function of number of targets and attentional demand (PO: pop-out, NPO: non-pop-out). Vertical bars represent the standard error of the mean. Figure 7b. Presentation time offset minus estimated search times expressed as a function of number of target items. Dashed lines illustrate linear fit (parameters reported in the main text).

Similarly to Experiment 3, participants were slower without than with perceptual pop-out, *F*(1, 9) = 145.4, *p*< .001, η² = .94. Presentation time again increased linearly with the number of targets that needed to be encoded into WM in both the pop-out and non pop-out conditions (linear fits were *R*^2^ = .976 for pop-out and *R*^2^ = .987 for non pop-out); these changes were highly significant, *F*(4, 36) = 66.6, *p*< .001, η² = .88 (see Figure 7a). As would be expected from the results of Experiment 3, the slope relating the average presentation time to the number of targets was steeper for non pop-out (681 ms) than for pop-out (229 ms), leading to a significant interaction between number of targets and attentional demand, *F*(4, 36) = 24.6, *p*< .001, η² = .73. The offset between the pop-out and non pop-out conditions increased gradually from 1721 ms, for one target, to 3563 ms, for five targets. In the pop-out conditions the presentation time did not differ significantly from those in Experiment 4, in which we did not use articulatory suppression *F*(1, 24) = 2.7, *p* = .11. Also, no difference was found when only the responses given in the most difficult condition (non pop-out with five targets) were investigated, *t*(24) = 1.38, *p* = .18.

Most importantly, the offsets in Experiment 5 did not differ significantly from those obtained in Experiment 3 (range 2145–3937 ms), *F*(1, 24) = 0.8, *p* = .37 (see Table 1). Next, we investigated the degree to which the presentation time offset between non pop-out and pop-out conditions could be explained by the model of repeated serial searches discussed and tested in Experiment 3. To this end, the amount of time spent on each target that could not be explained by the visual search by conducting analyses similar to those described in Experiment 3 was estimated. Thus, the estimated offsets in the serial search time (obtained in Experiment 2) were subtracted from the offsets in the presentation time obtained in the present experiment. To compare directly the results from the present experiment with those from Experiment 3, the results shown in Figure 7b also contain those from Figure 5b (Experiment 3). This comparison revealed a high similarity in the results. As in Experiment 3, again a large positive intercept of the resulting function was found, which indicates that with the lack of pop-out participants needed a constant time of 1269 ms irrespective of the number of targets and additional 216 ms to process each target item (slope 216 ms, intercept 1269 ms, linear fit *R*^2^ = .903). Neither the slopes nor the intercepts differed significantly from the corresponding ones obtained in Experiment 3, *t*(24) = 0.26, *p* = .797 for slope; *t*(24) = 0.25, *p* = .798 for intercept. Therefore, as across Experiments 1 and 4, the presentation time was also highly consistent in the case of Experiments 3 and 5.

These results indicate that memory for locations plays an important role in the present paradigm even when repeated searches for the relevant locations are prevented. The time needed to encode the shapes of complex objects into WM in the non pop-out condition corresponds closely to the sum of the time needed to encode the shapes in the pop-out condition and the time needed to memorize the locations of the targets. This behavioral evidence is highly consistent with the subjective reports on the two-step strategy obtained during the debriefing procedures in Experiments 1 and 3.

#### Reported encoding strategies

The majority of participants (9 of 10) reported using the same chunking strategy as described by the majority of the participants in Experiment 4.

## General discussion

The present study investigated whether and how participants can encode complex objects into WM while engaging spatial attention for a visual search task. Attentional demand and WM load was manipulated by changing either search efficiency in the visual search component of the task or the number of shapes to be encoded in the memory component of the task. Based on the participant-chosen presentation time we sought to isolate the processes participants used to perform the task successfully.

The data provided evidence for the two-step encoding strategy. In the non pop-out condition of Experiment 1, participants required a longer presentation time than would be expected based on the simple addition of the search time (as measured in Experiment 2), and the time needed for WM encoding. Experiment 3 ruled out that repeated searches of the same location could explain the additional costs on presentation time in the non pop-out condition. Experiments 4 and 5 demonstrated a close match between the times participants needed to memorize the locations only and the differences in the presentation time between pop-out and non pop-out conditions when participants needed to memorize the shapes of the targets. This match remained good across different memory loads even when repeated searches at relevant target locations were strongly reduced. These results were highly consistent with the participants’ subjective reports about the strategy that they used to achieve the objectives of the task.

It might be argued that other processes than those related to the memorizing of target locations contributed to the additional time cost in the non pop-out condition. WM suffers from a time-related decay as soon as attention is switched away and captured by concurrent activities (Barrouillet et al., 2007). Thus, the additional time cost in the non pop-out condition might also be related to an increased need to interleave the attention-demanding visual search with the maintenance of the already encoded shapes. This possibility was not directly tested in this study. However, our results suggest that the rehearsal of complex objects was more demanding than the rehearsal of locations. Therefore, it can be expected that the need to interleave the search with the maintenance should be higher when shapes, as compared with locations, needed to be memorized. Our findings did not support this prediction as the additional costs on presentation time in the non pop-out conditions were comparable across WM domains. Taken together, the experimental data, in combination with subjective reports, seemed to be most consistent with the two-step strategy that involved memorizing the locations of all the targets before memorizing the associated shapes.

Why would participants need to memorize target locations? One possible reason is that this is how they cope with the interference between WM and attention that would otherwise take place. Interference between selective attention and the storage of information in spatial WM has been well documented and interpreted in terms of common cognitive resources shared by these processes (Awh et al., 1998; Mayer et al., 2007; Oh & Kim, 2004; Smyth & Scholey, 1994; Woodman & Luck, 2004). The present findings suggest that interference between selective attention and WM encoding may not be restricted to the spatial domain, unlike the findings for WM maintenance (Oh & Kim, 2004; Woodman, Vogel, & Luck, 2001). Instead, it seems likely that in the non pop-out condition of the present experiment, interference occurred between the attentional resources needed for determination of the target locations (Treisman, 1998; Treisman & Gormican, 1988) and the WM resources needed to encode targets’ shapes.

What is the common mechanism required by the visual search and the encoding of object information into WM? Selective attention appears to be that mechanism. Representations of spatial locations are maintained in WM by keeping the spotlight of attention on these locations (Awh & Jonides, 2001; Awh et al., 1998). According to this account, selective attention is recruited in the service of a rehearsal-like function that keeps information active in WM and prevents its decay. A similar mechanism might come into play during WM encoding because of the necessity to verify the success of information transfer into WM, especially when multiple objects are presented simultaneously at different locations and need to be encoded. Another reason why selective attention should be involved both in the visual search and in WM encoding is related to the stimulus complexity. Complex objects, similar to those used in the present task, consist of multiple elementary features. Different features are bound into an integrated object through focused attention (Treisman & Gelade, 1980) and the storage of such information in WM requires capacity-limited attentional mechanisms as well (Wheeler & Treisman, 2002).

The implication of the present study is that the memory for locations may provide a coping mechanism for interference between search and memory. In the pop-out condition the unique elementary features attract the spotlight of attention by automatic bottom-up mechanisms (Treisman & Gelade, 1980). Along similar lines, the locations in the non pop-out condition, once memorized, might guide the attentional spotlight in an automatic-like fashion. Consistent with this notion, it has been proposed that in order to search for multiple targets efficiently, participants use spatial WM to keep track of identified targets (Horowitz & Wolfe, 2001).

It is possible that this storage of target locations was based on visual LTM because LTM is, in general, a tool for coping with capacity limitations. LTM is used during the chunking processes in WM (short-term memory) tasks (Chase & Simon, 1973; Cowan, 2001; Gobet et al., 2001; Miller, 1956) and is responsible for the development of skills and expertise in general (Chase & Ericsson, 1981; Hasher & Zacks, 1979; Shiffrin & Schneider, 1977). The main advantage of maintaining information in LTM, as opposed to WM, is that such storage does not seem to rely on capacity-limited resources (Ericsson & Kintsch, 1995; Phillips & Cristie, 1977). It has recently been shown that in a task similar to the present one, participants can readily store target locations into LTM when they need to memorize a number of locations that greatly exceeds the capacity of visual WM for such locations (Nikolić & Singer, 2007).

Real-life situations in which interference between WM and attention occurs may require similar coping mechanisms. One example of a cluttered visual scene, in which not only serial search but also other forms of spatial processing are needed, is map reading (e.g., Garden, Cornoldi, & Logie, 2002; Thorndike & Hayes-Roth, 1982). To find a suitable route, first the key locations (e.g., the origin and destination) need to be identified, and only can then the rest of the route be explored. If the route is non-trivial (multiple locations in-between and turns are involved), there might be at first interference between the memory for the examined part of the route and the search for the rest of the route. However, over time, as the route is being studied, knowledge will be acquired (including information about the sequence of landmarks along the route or about metric distances and angles that are integrated into a configural cognitive map), and the access to the route should gradually become easier. Similar processes should apply to other activities that involve visual WM and attention such as navigating through complex technical drawings or within one’s environment (Foo, Warren, Duchon, & Tarr, 2005; Garden et al., 2002; van Asselen, Fritschy, & Postma, 2006). In general, memory for locations might be the very mechanism that allows us to extract and encode relevant information from complex visual scenes when obvious cues that automatically draw attention are not available.

## Footnotes

1 The differences in performance between Experiments 1 and 3 should not be due to the articulatory suppression task used only in Experiment 3. This is because object naming would be the most likely advantage of verbalization and this could be used equally well in the pop-out and in the non pop-out condition. Therefore, participants would also have to be better in the pop-out condition of Experiment 3 compared with Experiment 1. However, it was found that in the pop-out condition the performance across these two experiments was identical. This was also the case in the most difficult condition (non pop-out with five targets) when investigated individually. Although articulatory suppression on verbal memory recall is not necessarily dramatic (Baddeley, 2000, 2003), this similarity in presentation time between Experiment 1 without articulatory suppression and Experiment 3 with articulatory suppression indicates that encoding was based, to a considerable degree, on visual processing.

## References

[R1] Alvarez G. A., Cavanagh P. (2004). The capacity of visual short-term memory is set both by visual
						information load and by number of objects.. Psychological Science.

[R2] Awh E., Jonides J. (2001). Overlapping mechanisms of attention and spatial working
						memory.. Trends in Cognitive Sciences.

[R3] Awh E., Jonides J., Reuter-Lorenz P. A. (1998). Rehearsal in spatial working memory.. Journal of Experimental Psychology: Human Perception and
						Performance.

[R4] Baddeley A. (2000). Working memory: Looking back and looking forward.. Nature Reviews Neuroscience.

[R5] Baddeley A. (2003). The episodic buffer: A new component of working
						memory?. Trends in Cognitive Science.

[R6] Barrouillet P., Bernardin S., Portrat S., Vergauwe E., Camos V. (2007). Time and cognitive load in working memory.. Journal of Experimental Psychology: Learning, Memory, and
						Cognition.

[R7] Besner D., Davies J., Daniels S. (1981). Reading for meaning: The effects of concurrent
						articulation.. Quarterly Journal of Experimental Psychology.

[R8] Cavanagh P., Alvarez G. A. (2005). Tracking multiple targets with multifocal
						attention.. Trends in Cognitive Science.

[R9] Chase W. G., Ericsson K. A., Anderson J. R. (1981). Skilled memory.. Cognitive skills and their acquisition.

[R10] Chase W. G., Simon H. A., Chase W. G. (1973). The mind’s eye in chess.. Visual information processing.

[R11] Corbetta M., Kincade J. M., Shulman G. L. (2002). Neural systems for visual orienting and their relationships to
						spatial working memory.. Journal of Cognitive Neuroscience.

[R12] Cowan N. (2001). The magical number 4 in short-term memory: A reconsideration of
						mental storage capacity.. Behavioral and Brain Sciences.

[R13] De Fockert J. W., Rees G., Frith C. D., Lavie N. (2001). The role of working memory in visual selective
						attention.. Science.

[R14] Duncan J. (1984). Selective attention and the organization of visual
						information.. Journal of Experimental Psychology: General.

[R15] Duncan J., Humphreys G. W. (1989). Visual search and stimulus similarity.. Psychological Review.

[R16] Duncan J., Ward R., Shapiro K. (1994). Direct measurement of attentional dwell time in human
						vision.. Nature.

[R17] Ericsson K. A., Kintsch W. (1995). Long-term working memory.. Psychological Review.

[R18] Foo P., Warren W. H., Duchon A., Tarr M. J. (2005). Do humans integrate routes into a cognitive map? Map- versus
						landmark-based navigation of novel shortcuts.. Journal of Experimental Psychology: Learning, Memory, and
						Cognition.

[R19] Garden S., Cornoldi C., Logie R. H. (2002). Visuo-spatial working memory in navigation.. Applied Cognitive Psychology.

[R20] Gobet F., Lane P. C., Croker S., Cheng P. C., Jones G., Oliver I., Pine J. M. (2001). Chunking mechanisms in human learning.. Trends in Cognitive Sciences.

[R21] Hasher L., Zacks R. T. (1979). Automatic and effortful processes in memory.. Journal of Experimental Psychology: General.

[R22] Horowitz T. S., Wolfe J. M. (2001). Search for multiple targets: Remember the targets, forget the
						search.. Perception & Psychophysics.

[R23] Irwin D. E. (1992). Memory for position and identity across eye
						movements.. Journal of Experimental Psychology: Learning, Memory, and
						Cognition.

[R24] Jolicœur P., Dell’Acqua R. (1998). The demonstration of short-term consolidation.. Cognitive Psychology.

[R25] Jolicœur P., Dell’Acqua R. (1999). Attentional and structural constraints on visual
						encoding.. Psychological Research.

[R26] LaBar K. S., Gitelman D. R., Parrish T. B., Mesulam M. M. (1999). Neuroanatomic overlap of working memory and spatial attention
						networks: A functional MRI comparison within subjects.. NeuroImage.

[R27] Lavie N., Hirst A., de Fockert J. W., Viding E. (2004). Load theory of selective attention and cognitive
						control.. Journal of Experimental Psychology: General.

[R28] Luck S. J., Vogel E. K. (1997). The capacity of visual working memory for features and
						conjunctions.. Nature.

[R29] Mayer J. S., Bittner R. A., Nikolić D., Bledowski C., Goebel R., Linden D. E. J. (2007). Common neural substrates for visual working memory and
						attention.. NeuroImage.

[R30] Miller G. A. (1956). The magic number seven plus or minus two: Some limits on our
						capacity for processing information.. Psychological Review.

[R31] Murray D. J. (1968). Articulation and acoustic confusability in short-term
						memory.. Journal of Experimental Psychology.

[R32] Nikolić D., Singer W. (2007). Creation of visual long-term memory.. Perception & Psychophysics.

[R33] Oh S. H., Kim M. S. (2004). The role of spatial working memory in visual search
						efficiency.. Psychonomic Bulletin & Review.

[R34] Olsson H., Poom L. (2005). Visual memory needs categories.. Proceedings of the National Academy of Sciences of the United States of
						America.

[R35] Pashler H. (1988). Familiarity and visual change detection.. Perception & Psychophysics.

[R36] Peterson M. S., Kramer A. F., Wang R. F., Irwin D. E., McCarley J. S. (2001). Visual search has memory.. Psychological Science.

[R37] Phillips W. A. (1974). On the distinction between sensory storage and short-term visual
						memory.. Perception & Psychophysics.

[R38] Phillips W. A., Christie D. F. M. (1977). Components of visual memory.. Quarterly Journal of Experimental Psychology.

[R39] Pollmann S., von Cramon D. Y. (2000). Object working memory and visuospatial processing: Functional
						neuroanatomy analyzed by event-related fMRI.. Experimental Brain Research.

[R40] Pylyshyn Z. W., Storm R. W. (1988). Tracking multiple independent targets: Evidence for a parallel
						tracking mechanism.. Spatial Vision.

[R41] Scholl B. J. (2001). Objects and attention: The state of the art.. Cognition.

[R42] Shiffrin R. M., Schneider W. (1977). Controlled and automatic human information processing: II.
						Perceptual learning, automatic attending, and a general
						theory.. Psychological Review.

[R43] Smyth M. M., Scholey K. A. (1994). Interference in immediate spatial memory.. Memory & Cognition.

[R44] Thorndike P. W., Hayes-Roth B. (1982). Differences in spatial knowledge acquired from maps and
						navigation.. Cognitive Psychology.

[R45] Treisman A. (1998). Feature binding, attention, and object
						perception.. Philosophical Transactions of the Royal Society of London, B, Biological
						Sciences.

[R46] Treisman A., Gelade G. (1980). A feature-integration theory of attention.. Cognitive Psychology.

[R47] Treisman A., Gormican S. (1988). Feature analysis in early vision: Evidence from search
						asymmetries.. Psychological Review.

[R48] Treisman A., Sato S. (1990). Conjunction search revisited.. Journal of Experimental Psychology: Human Perception and
						Performance.

[R49] Van Asselen M., Fritschy E., Postma A. (2006). The influence of intentional and incidental learning on acquiring
						spatial knowledge during navigation.. Psychological Research.

[R50] Vecera S. P., Farah M. J. (1994). Does visual attention select objects or
						locations?. Journal of Experimental Psychology: General.

[R51] Wheeler M. E., Treisman A. M. (2002). Binding in short-term visual memory.. Journal of Experimental Psychology: General.

[R52] Wolfe J. M., Pashler H. (1998a). Visual search. Attention.

[R53] Wolfe J. M. (1998b). What can 1 million trials tell us about visual
						search?. Psychological Science.

[R54] Woodman G. F., Luck S. J. (2004). Visual search is slowed when visuospatial working memory is
						occupied.. Psychonomic Bulletin & Review.

[R55] Woodman G. F., Vogel E. K., Luck S. J. (2001). Visual search remains efficient when visual working memory is
						full.. Psychological Science.

